# Neuro-mechanical determinants of repeated treadmill sprints - Usefulness of an “hypoxic to normoxic recovery” approach

**DOI:** 10.3389/fphys.2015.00260

**Published:** 2015-09-23

**Authors:** Olivier Girard, Franck Brocherie, Jean-Benoit Morin, Grégoire P. Millet

**Affiliations:** ^1^Department of Physiology, Faculty of Biology and Medicine, Institute of Sport Sciences, University of LausanneLausanne, Switzerland; ^2^Athlete Health and Performance Research Center, Aspetar, Qatar Orthopaedic and Sports Medicine HospitalDoha, Qatar; ^3^Laboratory of Human Motricity, Education Sport and Health, University of Nice Sophia AntipolisNice, France

**Keywords:** repeated-sprint ability, running mechanics, hypoxia, electromyography, recovery

## Abstract

To improve our understanding of the limiting factors during repeated sprinting, we manipulated hypoxia severity during an initial set and examined the effects on performance and associated neuro-mechanical alterations during a subsequent set performed in normoxia. On separate days, 13 active males performed eight 5-s sprints (recovery = 25 s) on an instrumented treadmill in either normoxia near sea-level (SL; FiO_2_ = 20.9%), moderate (MH; FiO_2_ = 16.8%) or severe normobaric hypoxia (SH; FiO_2_ = 13.3%) followed, 6 min later, by four 5-s sprints (recovery = 25 s) in normoxia. Throughout the first set, along with distance covered [larger sprint decrement score in SH (−8.2%) compared to SL (−5.3%) and MH (−7.2%); *P* < 0.05], changes in contact time, step frequency and root mean square activity (surface electromyography) of the quadriceps (*Rectus femoris* muscle) in SH exceeded those in SL and MH (*P* < 0.05). During first sprint of the subsequent normoxic set, the distance covered (99.6, 96.4, and 98.3% of sprint 1 in SL, MH, and SH, respectively), the main kinetic (mean vertical, horizontal, and resultant forces) and kinematic (contact time and step frequency) variables as well as surface electromyogram of quadriceps and plantar flexor muscles were fully recovered, with no significant difference between conditions. Despite differing hypoxic severity levels during sprints 1–8, performance and neuro-mechanical patterns did not differ during the four sprints of the second set performed in normoxia. In summary, under the circumstances of this study (participant background, exercise-to-rest ratio, hypoxia exposure), sprint mechanical performance and neural alterations were largely influenced by the hypoxia severity in an initial set of repeated sprints. However, hypoxia had no residual effect during a subsequent set performed in normoxia. Hence, the recovery of performance and associated neuro-mechanical alterations was complete after resting for 6 min near sea level, with a similar fatigue pattern across conditions during subsequent repeated sprints in normoxia.

## Introduction

Intense physical efforts performed at or near maximal speeds are often crucial for successful participation in intermittent sports (e.g., team or racket sports). For instance, top-level soccer players complete more high-intensity running or sprinting than their lower-level counterparts (Mohr et al., 2003, 2008). However, irrespectively of competitive standard, the volume of all high-intensity actions decline over the course of a game, reflecting muscle fatigue development (Mohr et al., 2008). Although, still debated (Carling, 2013), the repeated-sprint ability (RSA) is commonly viewed as an important marker of successful physical performance in these disciplines.

While RSA has been increasingly investigated over the last decade, to date, most of the available studies focused only on the physiological features of this fitness component. Evaluation of the biomechanical aspects of running RSA have insofar been limited to either indirect measures of stride characteristics (i.e., pressure insoles) (Girard et al., 2011a; Brocherie et al., 2015) or direct sprint kinetics/kinematics assessments (i.e., force platforms), but only for a discrete number of steps at various intervals during the sprint distance (Girard et al., 2011b). Using instrumented, sprint treadmills makes now possible to deepen our knowledge about the biomechanical manifestation of fatigue during repeated sprinting (Morin et al., 2011). For instance, through direct measurement of ground reaction forces, Girard et al. (2015a) reported significant decrease in propulsive power and step frequency with fatigue while contact time and step length increased, when five maximal 5-s sprints with incomplete recoveries (25 s) were repeated.

Peripheral mechanisms, that include limitation in energy supply and the intramuscular accumulation of metabolic by-products, have been traditionally associated to fatigue development during repeated sprinting (Girard et al., 2011c). Consideration of neural factors (i.e., neural drive and muscle recruitment strategies) as significant contributors to fatigue etiology during RSA protocols stem from parallel reductions in amplitude of quadriceps surface electromyography (EMG) signals (i.e., a reasonable proxy for net motor unit activity) and in sprint performance (Mendez-Villanueva et al., 2008; Billaut et al., 2013; Bowtell et al., 2014; Brocherie et al., 2015). For instance, Brocherie et al. (2015) demonstrated a disproportionate decrease in motor unit recruitment inferred via EMG signaling [Root Mean Square (RMS) activity] of *Rectus femoris* and *Biceps femoris* muscles over sprint times when professional football players completed the repeated anaerobic sprint test on artificial turf. Although, muscle activation capacity of plantar flexors decreases from pre- to post-RSA running (Perrey et al., 2010), the question of whether this muscle group is subjected to similar neural adjustments than those seen for the quadriceps during actual sprint repetitions remains undetermined.

When attempting to evaluate RSA and its fatigue-causing factors, a single set of a fixed number of 5–15 sprints (i.e., usually of 5–10 s) with (incomplete) recovery of less than 30 s (i.e., usually passive) has most commonly been used (Girard et al., 2011c). Admittedly, while valuable knowledge on how fatigue manifests and the potential contribution of neural factors can be gained from such RSA tests' format, derived information remains mainly descriptive. Innovative analysis methods that are based on the comparison of fatigue responses and recovery of performance during and between sets of repeated sprints, respectively, have emerged (Girard et al., 2015b). By linking the aforementioned changes to muscle metabolism and neuromuscular function, such approaches support the idea that previous repeated-sprint exercise has a negative “carry-over” impact on physiological strain, perception of effort and performance during the next bout of activity (Mendez-Villanueva et al., 2007, 2012; Billaut et al., 2013). With this in mind, it is surprising that little attention has been directed toward the usefulness of the “recovery of performance approach” to shed more light on how running mechanics and muscle activation patterns are altered during RSA run-based tests.

Extreme environments such as hypoxia [i.e., a reduction in environmental oxygen (O_2_) availability] are known to lead to premature fatigue and exacerbated cardiorespiratory and perceptual responses during repeated-sprint exercise (Billaut et al., 2013; Bowtell et al., 2014; Goods et al., 2014). By majoring RSA-induced demands (and thereby recovery requirements) on the neuromuscular system during an initial set (i.e., larger changes within the central nervous system with severer hypoxic levels), it seems reasonable to speculate that performance decrement during a subsequent repeated-sprint exercise would be exacerbated. Accordingly, modifying the ensuing recovery rate of repeated-sprint performance from previous strenuous exercise highlights a context whereby neuro-mechanical determinants of RSA running performance could be explored from a new perspective (Minett and Duffield, 2014).

Our intention was therefore to manipulate hypoxia severity during an initial repeated-sprint set and examine the effect on sprinting performance, running mechanics (kinetics and kinematics) and lower-limbs neuromuscular activity (surface EMG activity) during a subsequent set performed in normoxia. We hypothesized that, with severer hypoxia levels during a first repeated-sprint set expected to major RSA-induced demands placed on the neuromuscular system, larger recovery requirements and fatigue-related residual or “carry-over” effects from the previous set would, in turn, negatively influence fatigability during the completion of a second set performed in normoxia.

## Methods

### Subjects

Thirteen male recreational team- (i.e., football, rugby, basketball) and racket- (i.e., tennis, squash) sport players (Mean ± SD: 31.2 ± 4.8 years; 178.4 ± 6.6 cm; 74.3 ± 8.2 kg) participated in the study. In the 6 months preceding the study, subjects trained on average 4.5 ± 2.5 h.wk^−1^, which included activity-specific training (i.e., technical and tactical skills), aerobic and anaerobic training (i.e., on- and off-court/field exercises) and basic strength training. Although, training content of the tested athletes largely focused on accelerated runs, their sprinting skills are deemed to be “moderate” compared to “elite” (i.e., national to international level) sprinters (Rabita et al., 2015) and/or team-sport athletes (Brocherie et al., 2015). All subjects were born and raised at < 1000 m and had not traveled to elevations >1000 m in the 3 months prior to investigation. They gave their informed, written consent preceding the commencement of the experiment. Experimental protocol was conducted according to the Declaration of Helsinki for use of Human Subjects and approved by the Ethics Committee of *Shafallah Medical Genetics Center*.

### Experimental procedure

About 1 week prior to testing, subjects undertook a complete preliminary session where they performed short (<5 s) “familiarization” treadmill sprints at increasing intensities while wearing a facemask for habituation (i.e., the hypoxic system was turned off at this occasion), with full recovery and until being comfortable with treadmill maximal sprint technique (which generally required 7–10 trials). Subjects then performed three maximal 5-s single sprints separated by 2 min of passive rest. All participants satisfied the criteria of having a coefficient of variation < 2.2% for distance covered across three successive trials (Girard et al., 2015c). After 10 min of rest, the complete RSA test was completed. Strong verbal encouragement was given during all maximal efforts.

Subjects then came to the laboratory (well-ventilated at a constant temperature of ~25°C and 40% relative humidity) for three experimental sessions (~1 h; counterbalanced randomized crossover design in double-blind fashion), with at least 3–4 days apart, including a repeated-sprint running protocol on a treadmill sprint ergometer. They performed their trials at the same time of the day (±1 h) and wore similar sports gear (running shoes, short, and T-shirt). They were instructed to maintain their normal diet (i.e., avoiding any nutritional supplements or alcohol consumption), sleeping (i.e., ≥7 h/night) and training (i.e., avoiding vigorous exercise 24 h before every trial) habits during the 1–2 weeks period of testing to prevent any possible interference on their sprinting abilities. Subjects were instructed to drink 4–6 mL of water per kilogram of body mass every 2.5 h on the day before each experimental session to ensure euhydration at the start of exercise. They were permitted to drink *ad libitum* during the warm-up procedure.

### Repeated-sprint exercise protocol

The exercise protocol consisted of performing first eight, 5-s “all-out” sprints interspersed with 25 s of passive rest and randomly conducted near sea level (SL; FiO_2_ ~20.9%), at moderate and severe simulated altitudes (normobaric hypoxia) of 1800 m (MH; FiO_2_ ~16.6%) and 3600 m (SH; FiO_2_ ~13.0%), respectively. This was followed, after 6 min of passive rest (i.e., subjects breathed ambient air), by four, 5-s “all-out” sprints also interspersed by 25 s of passive rest but always performed at SL. During recovery periods, subjects stood on the treadmill. Before all tests, subjects completed a standardized warm-up (i.e., on the instrumented treadmill with subjects breathing ambient air) consisting of 10 min of running at 10 km.h^−1^, followed by 15 min of sprint-specific muscular warm-up exercises [i.e., 3 × (high knee, high heels, butt-kick, skipping for ~10 s with 30-s walking in between), followed by 3 × (3 steps accelerations at a subjective “sense of effort” of 7, 8, and 9), then by 2 × (3-s sprints at a subjective “sense of effort” of 8 and 9] (Christian et al., 2014). Afterwards, three maximal 5-s single sprints (i.e., the best of these three trials was used as the criterion score), separated by 2 min of passive rest, were completed. Finally, after a facemask connected to a portable hypoxic generator has been attached on subjects, they were allowed 5-min of free cool down prior to the repeated-sprint protocol. Testing protocols were run in a double-blind fashion in that subjects and one investigator were blinded toward the environmental condition of the initial set. The efficacy of the subjects' blinding procedure was evaluated after each experimental session by questionnaires in which subjects were asked whether they believed to be exercising at SL, MH, or SH. We are confident that the blinding procedure was efficient, as only four athletes were able to correctly identify the order of treatment.

### Altitude simulation

Normobaric hypoxia was obtained by mixing nitrogen into ambient air under control of FiO_2_ (Altitrainer, SMTec SA, Nyon, Switzerland). This gas-mixing system enriches the inspired air by adding a fixed quantity of nitrogen via a 30 L mixing chamber, with the dilution being constantly controlled by a PO_2_ probe (with a precision of ± 0.82 Torr and safety set at FiO_2_ = 9.7%). This device allows the production of large quantities of a hypoxic gas mixture (up to 200 L.min^−1^), with an easily adjustable O_2_ fraction over a large range, and a short response time (between 15 and 45 s), expressed either by the equivalent altitude or by the O_2_ partial pressure, taking into account the barometric pressure. For blinding purposes, subjects who always breathed through the same set-up (also in normoxia), inhaled the mixture contained in the buffer tank through a Hans Rudolph two-way respiratory valve. Subjects were instrumented with the facemask 5 min before the repeated-sprint exercise (i.e., after the three “reference” sprints at warm-up termination) until the end of the first set of eight sprints.

### Instrumented sprint treadmill

The sprints were performed on an instrumented motorized treadmill (ADAL3D-WR, Medical Development—HEF Tecmachine, Andrézieux-Bouthéon, France). Briefly, it is mounted on a highly rigid metal frame fixed to the ground through four piezoelectric force transducers (KI 9077b; Kistler, Winterthur, Switzerland) and installed on a specially engineered concrete slab to ensure maximal rigidity of the supporting ground. This motorized treadmill allows subjects to sprint and produce realistic acceleration and high running velocities (Morin et al., 2010). A single-pass waist and a stiff rope (1 cm in diameter, ~2 m length) were used to tether subjects to the 0.4-m vertical rail anchored to the wall behind them. When correctly attached, subjects were required to lean forward in a typical and standardized crouched sprint-start position with their left foot forward. This starting position was used and standardized all along the sprint series. After a 5-s countdown (“5 s, 3-2-1-Go” given by both visual and audio instructions by the same investigator), the treadmill was released, and the belt began to accelerate as subjects applied a positive horizontal force.

### Mechanical variables

Data were continuously sampled at 1000 Hz over the sprints, and after appropriate filtering (Butterworth-type 30 Hz low-pass filter), instantaneous data of vertical, net horizontal and total (i.e., resultant) ground reaction forces were averaged for each support phase (vertical force above 30 N) over the 5-s sprints, and expressed in body weight (BW). These data were completed by measurements of the main step kinematic variables: contact time, aerial time, step frequency, and step length.

### Electromyography

EMG signals from superficial *Rectus femoris, Vastus lateralis, Biceps femoris, Gastrocnemius medialis, Gastrocnemius lateralis*, and *Tibialis anterior* of the right lower limb were recorded using pre-amplified bi-polar surface EMG (Delsys, Trigno Wireless, Boston, Massachusetts, USA) with an inter-electrode (center-to-center) distance of 20 mm and placed according to the surface electromyography for the non-invasive assessment of muscles (SENIAM) project's recommendations. Before electrode placement, the skin was lightly abraded and washed to remove surface layers of dead skin, hair, and oil. All electrodes were secured with elastic cohesive bandage to reduce any movement of electrodes during sprinting or artifact in the signal. The position of the EMG electrodes was marked with indelible ink to ensure that they were placed in the same location during subsequent visits. The myoelectric signal was amplified (gain = 1000×) and filtered (bandwidth frequency = 20–450 Hz) to minimize extraneous noise and possible movement artifacts in the low-frequency region and to eliminate aliasing and other artifacts in the high-frequency region. Surface EMG signals were recorded continuously during each 5-s sprint with a sampling frequency of 2000 Hz using a dedicated acquisition system (CED 1401, Cambridge Electronic Design, Cambridge, UK) and analyzed offline (Spike2 v3.21; Cambridge Electronic Design, Cambridge, UK). The activity of each muscle was determined by measuring the mean value of the RMS signal between the onset and the end of the burst for each 5-s sprint. For each individual, a burst of muscle activity was identified as the amplitude of muscle activity exceeding 15% of peak activation for more than 10% of the stride (Brocherie et al., 2015). To investigate the difference in EMG frequency between the three conditions, the filtered EMG data from each sprint were further transformed into the frequency domain using a fast Fourier transformation and the median power frequency (MPF) of the resulting power spectrum density was calculated (Matsuura et al., 2006). The RMS and MPF were normalized to the first sprint value of each condition, which was assigned the value of 100% (Mendez-Villanueva et al., 2012; Brocherie et al., 2015).

### Responses to exercise

Heart rate (HR) and pulse O_2_ saturation (SpO_2_) were monitored and estimated, respectively, via a Polar transmitter-receiver (Wearlink T-31, Polar Electro Oy, Kempele, Finland) and non-invasive pulse oximetry using a finger probe (Palmsat 2500, NONIN Medical Inc., Plymouth, MI, USA). The subjects were unable to view any of the HR or SpO_2_ values since receivers were attached on the handrails of the treadmill facing one experimenter. Together with HR and SpO_2_, ratings of perceived exertion (RPE) were recorded using the Borg 6–20 scale (i.e., 6 = no exertion at all, 20 = maximal exertion) exactly 10 s following each sprint (i.e., peak values likely to be obtained), where subjects were instructed to reflect on their perception of overall peripheral discomfort during the preceding exercise bout (Christian et al., 2014). In addition, SpO_2_ was recorded between before the warm-up and 4 min into recovery between the two repeated-sprint sets. A capillary blood sample was taken from the fingertip and analyzed for blood lactate concentration with a portable analyzer (Lactate Pro LT-1710, Arkray, Japan) before the warm-up, 2 min after the first set of 8 sprints and 2 min after the second set of 4 sprints.

### Data analysis and statistics

Subjects completed between 15 and 18 steps during each 5-s sprint. After excluding the last two ground contacts, the remaining three consecutive steps were used for final analysis of sprint kinetics/kinematics (Brocherie et al., 2015). While subjects performed a total of 12 sprints, only responses to exercise, running mechanical and surface EMG data collected for sprint number 1, 4, 8, 9, and 12 were considered for the main analysis. For the main running mechanical variables, the average of sprints number 1–4, 5–8, and 9–12 have also been compared.

Values are expressed as mean ± SD. Two-Way repeated-measures analysis of variance (ANOVAs) [Time (Sprints 1, 4, 8, 9, and 12 or Sprints number 1–4, 5–8, and 9–12) × Condition (SL, MH, and SH)] were used to compare investigated variables. To assess assumptions of variance, Mauchly's test of sphericity was performed using all ANOVA results. A Greenhouse-Geisser correction was performed to adjust the degree of freedom if an assumption was violated, while a Bonferroni *post-hoc* multiple comparison was performed if a significant main effect was observed. For each ANOVA, partial eta-squared was calculated as measures of effect size. Values of 0.01, 0.06, and above 0.14 were considered as small, medium, and large, respectively. All statistical calculations were performed using SPSS statistical software V.21.0 (IBM Corp., Armonk, NY, USA). The significance level was set at *P* < 0.05.

## Results

### Responses to exercise

**Figure 1:**
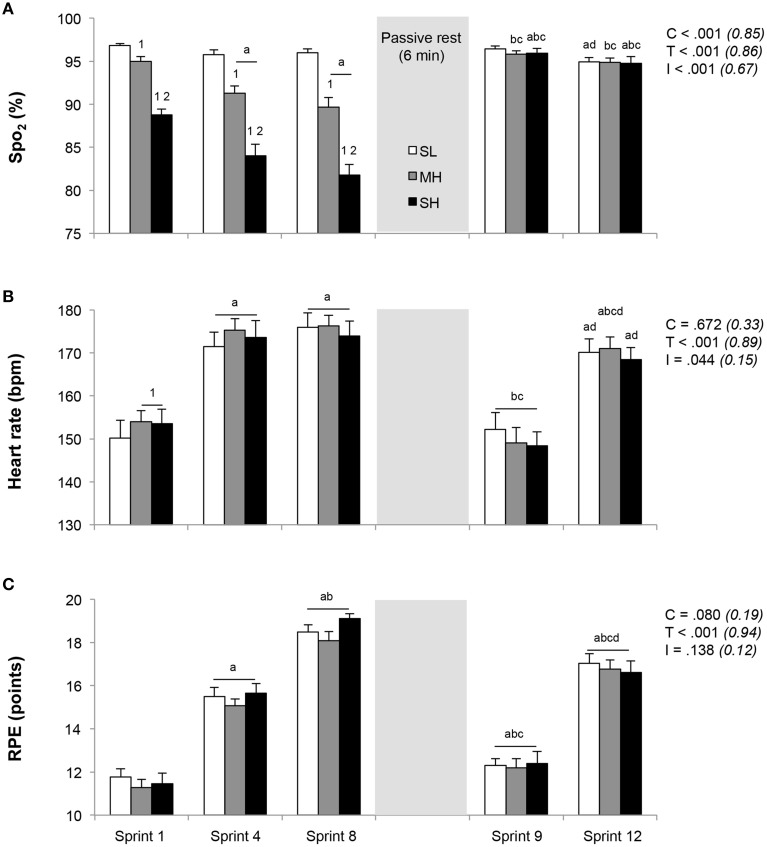
**Changes in exercise responses (A, SpO_2_; B, heart rate; C, RPE)**. Mean ± SD (*n* = 13). The repeated-sprint exercise protocol included a first set of eight sprints performed at sea level (SL), moderate (MH), or severe hypoxia (SH), while the second set of four sprints was always performed at SL. SpO_2_, arterial oxygen saturation (estimated by pulse oxymetry); RPE, rating of perceived exertion. C, T, and I, respectively refer to ANOVA main effects of condition, time, and interaction between these two factors with *P*-value and partial eta-squared into brackets. ^a^, ^b^, ^c^, and ^d^ significantly different from sprint 1, 4, 8, and 9, respectively (*P* < 0.05). ^1^ and ^2^ significantly different from SL and MH, respectively (*P* < 0.05).

Responses to exercise across the three conditions are depicted in Figure 1. During the initial set of sprints, SpO_2_ was significantly reduced for each simulated altitude ascent (*P* < 0.05). Lower SpO_2_ values were recorded for both sprints 4 and 8 (no difference) vs. sprint 1 in MH and SH, while no change occurred at SL. In response to sprint 1, HR was significantly higher in MH and SH compared to SL (*P* < 0.05), while RPE values were similar. Both HR and RPE increased significantly from sprint 1–4 (*P* < 0.05), while only RPE further increased at sprint 8 in reference to sprint 4 (*P* < 0.05), yet with similar values across conditions. Compared to prior to the warm-up (96.9 ± 0.4%), SpO_2_ were not different among conditions 4 min into recovery between the two repeated-sprint sets (96.2 ± 0.5%; all conditions compounded, *P*>0.05). During sprint 9, after 6 min of rest, SpO_2_, HR, and RPE values recovered significantly in relation to those achieved in sprint 4 and 8 (*P* < 0.05), with no difference between conditions. Whereas RPE values remained elevated compared to those measured in response to sprint 1, HR values recorded after sprint 1 and 9 were not different. At sprint 12, HR, and RPE values did not differ between conditions, while RPE was larger than in sprint 4 (*P* < 0.05).

From pre- to + 2 min post-set 1, the execution of the initial set of 8 sprints resulted in similar increases in blood lactate concentration (SL: 1.4 ± 0.4 vs. 9.9 ± 1.7 mmol.L^−1^, MH: 1.4 ± 0.4 vs. 10.4 ± 1.8 mmol.L^−1^, and SH: 1.4 ± 0.4 vs. 10.7 ± 2.1 mmol.L^−1^; *P* < 0.001), irrespectively of the environmental condition. There was a further global increase of blood lactate concentration values (10.8 ± 1.9, 11.2 ± 1.7 and 10.6 ± 2.2 mmol.L^−1^ in SL, MH, and SH, respectively; P < 0.05) recorded +2 min post-set 2 (i.e., after the completion of 4 additional normoxic sprints) in reference to post-set 1.

### Sprint performance and running kinetics

**Figure 2:**
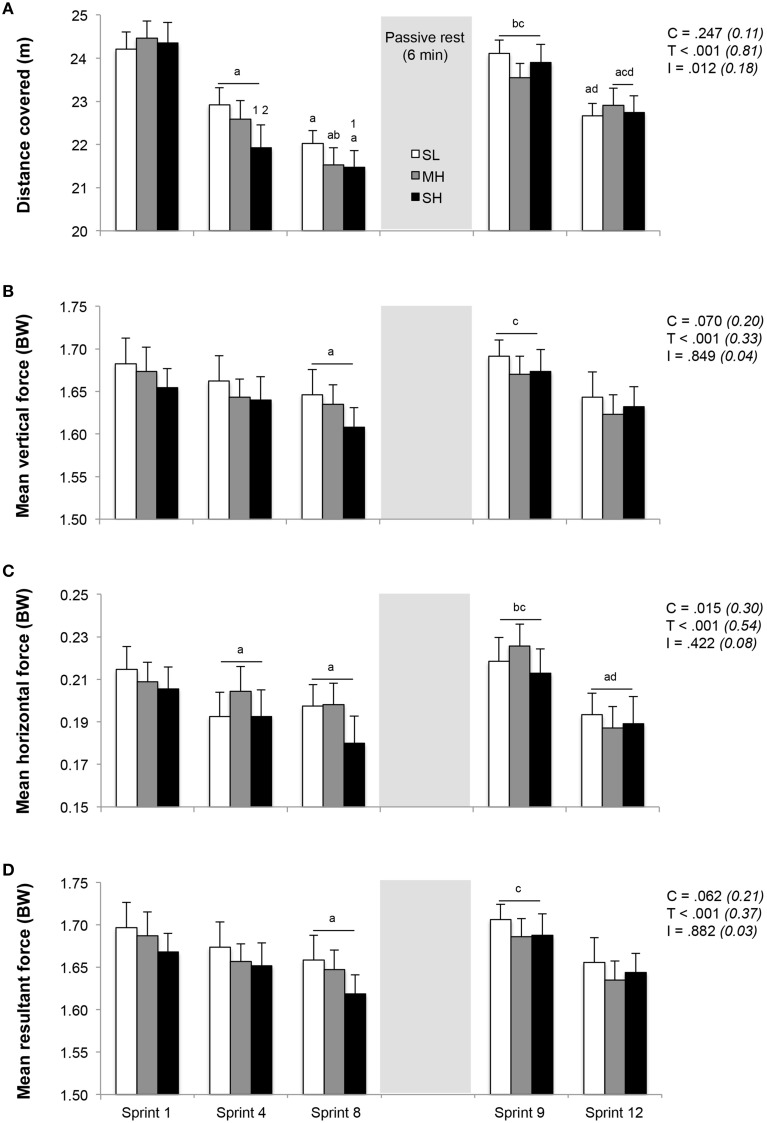
**Changes in distance covered (A) and stride kinetics (B, mean vertical force; C, mean horizontal force; D, mean resultant force)**. Mean ± SD (*n* = 13). The repeated-sprint exercise protocol included a first set of eight sprints performed at sea level (SL), moderate (MH), or severe hypoxia (SH), while the second set of four sprints was always performed at SL. C, T, and I, respectively refer to ANOVA main effects of condition, time and interaction between these two factors with *P*-value and partial eta-squared into brackets. ^a^, ^b^, ^c^, and ^d^ significantly different from sprint 1, 4, 8, and 9, respectively (*P* < 0.05). ^1^ and ^2^ significant different from SL and MH, respectively (*P* < 0.05).

Distance ran and associated running kinetics during the repeated-sprint exercise are displayed in Figure 2. No difference was found in distance ran during the first sprint between SL, MH and SH (24.2 ± 1.4, 24.1 ± 1.5, and 24.2 ± 2.0 m, respectively). However, sprint performance decreased to a larger extent in SH compared to SL, as evidenced by larger reductions in distance ran during sprint 4 (−9.9±5.2% vs. −5.3±2.8%; *P* < 0.05) and 8 (−11.7±5.2% vs. −8.9±4.1%; *P* < 0.05) in reference to sprint 1. Horizontal, but not vertical and total forces, significantly decreased from sprint 1 to 4 (*P* < 0.05). During sprint 8, values for vertical, horizontal and total forces were significantly lower (all conditions pooled; −2.3±1.9%, −8.6±6.5%, and −2.4±1.9%, respectively; *P* < 0.05) in reference to sprint 1.

During sprint 9, following 6 min of rest, sprint performance and running kinetics recovered significantly, as evidenced by larger values compared to those reached during sprint 8 (distance ran and horizontal forces; *P* < 0.05). Sprint 9 values did not differ from those achieved during sprint 1 and were similar between conditions. Over the last 4 sprints (sprint 9–12), distance ran and horizontal forces decreased similarly by an average of −4.5±2.5% and −13.1±9.6% (all conditions pooled; *P* < 0.05), while the decrease in vertical forces (−2.6±4.9%) and total forces (−2.8±4.9%) were not significant.

### Running kinematics

**Figure 3:**
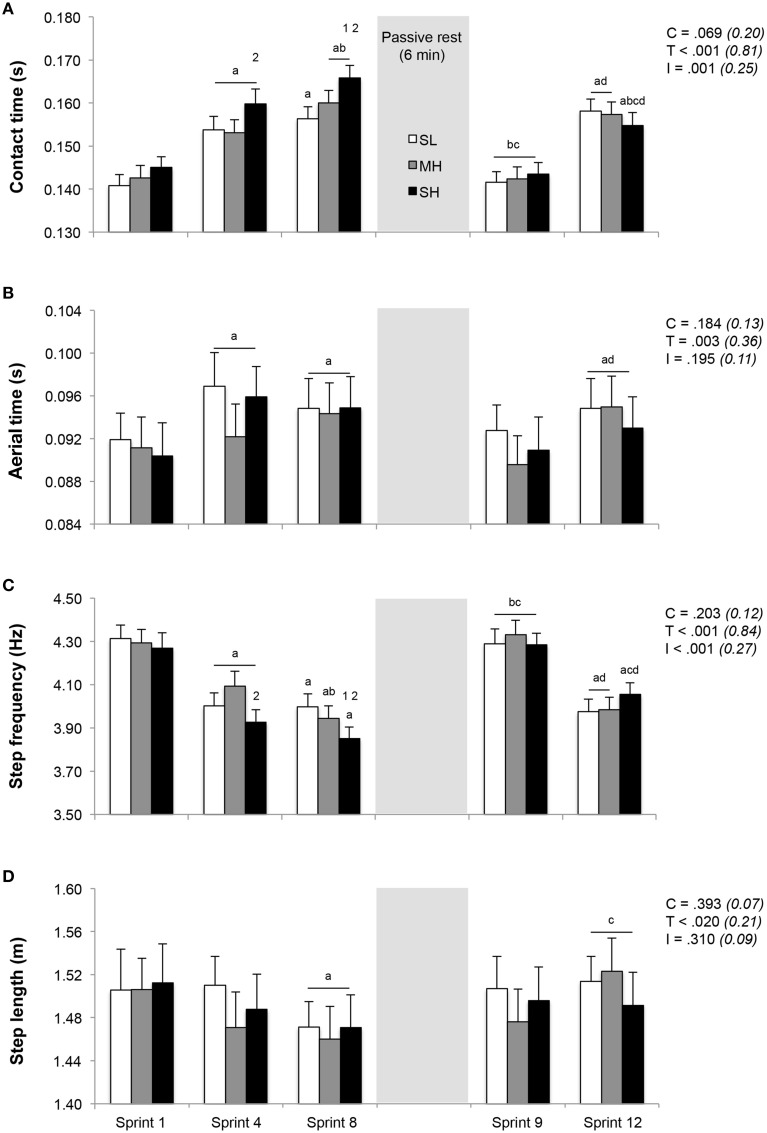
**Changes in stride kinematics (A, contact time; B, aerial time; C, step frequency; D, step length)**. Mean ± SD (*n* = 13). The repeated-sprint exercise protocol included a first set of eight sprints performed at sea level (SL), moderate (MH) or severe hypoxia (SH), while the second set of four sprints was always performed at SL. C, T, and I, respectively refer to ANOVA main effects of condition, time and interaction between these two factors with *P*-value and partial eta-squared into brackets. ^a^, ^b^, ^c^, and ^d^ significantly different from sprint 1, 4, 8, and 9, respectively (*P* < 0.05). ^1^ and ^2^ significant different from SL and MH, respectively (*P* < 0.05).

Running kinematics across the repeated sprints are displayed in Figure 3. Whereas step length remained unchanged, both contact and aerial times lengthened and step frequency decreased from sprint 1 to 4. During sprint 4, the increase in contact time and the decrease in step frequency were significantly larger in SH compared to MH (*P* < 0.05). From sprint 1 to sprint 8, the increase in contact time (+14.5 ± 6.1% vs. +11.2 ± 6.8% and +12.4 ± 5.1%; *P* < 0.05) and decrease in step frequency (−9.7±4.2% vs. −7.2±3.7% and −8.1±2.7%; *P* < 0.05) were larger in SH compared to SL and MH. Independently of the condition, aerial time lengthened (+4.2 ± 2.9%; *P* < 0.05) and step length decreased (−2.5±3.0%; *P* < 0.05) from sprint 1 to 8. After 6 min of rest between sprints 8 and 9, sprint kinematic values during sprint 9 were not statistically different from those recorded during sprint 1, with also no significant difference between conditions. During subsequent sprints (9–12), irrespectively of the condition, contact time (+10.2 ± 5.2%) and aerial time (+3.8 ± 3.3%) lengthened (*P* < 0.05), step frequency (−6.2±2.6%) decreased (*P* < 0.05) and step length (+1.2 ± 3.0%) remained unchanged.

Compared to SL and MH, contact time and step frequency values corresponding to the average of sprints 1 to 4 and sprints 5 to 8 differed under SH (Table 1; *P* < 0.05). The averaged values of distance covered, kinetics and kinematics for sprints 9–12 were similar across conditions and were not statistically different than the average of sprints 1–4.

**Table 1:** **Sprint performance, kinetics, and kinematics averaged for sprints 1–4, 5–8, and 9–12**.

**Variables**	**Average of sprints**			**ANOVA *p*-value (partial eta-squared)**		
	**1–4**	**5–8**	**9–12**	**Condition**	**Time**	**Interaction**
**DISTANCE (M)**						
SL	23.48±1.33	22.32±1.06	23.32±1.53	<0.001	0.091	0.119
MH	23.31±1.36	22.04±1.32	23.24±1.36	*(0.81)*	*(0.18)*	*(0.14)*
SH	22.92±1.51	21.71±1.27	23.30±1.29			
**VERTICAL FORCE (BW)**						
SL	1.67±0.09	1.65±0.09	1.67±0.10	0.003	0.208	0.292
MH	1.67±0.09	1.64±0.08	1.65±0.09	*(0.39)*	*(0.12)*	*(0.10)*
SH	1.66±0.08	1.63±0.09	1.66±0.09			
**HORIZONTAL FORCE (BW)**						
SL	0.20±0.04	0.20±0.04	0.20±0.04	0.004	0.013	0.418
MH	0.21±0.04	0.20±0.03	0.20±0.04	*(0.37)*	*(0.31)*	*(0.07)*
SH	0.20±0.04	0.18±0.04	0.20±0.04			
**RESULTANT FORCES (BW)**						
SL	1.69±0.09	1.66±0.09	1.68±0.10	0.003	0.178	0.385
MH	1.68±0.09	1.65±0.08	1.67±0.09	*(0.38)*	*(0.13)*	*(0.08)*
SH	1.67±0.08	1.64±0.09	1.68±0.09			
**CONTACT TIME (S)**						
SL	0.148±0.010	0.155±0.008^a^	0.151±0.011	<0.001	0.015	<0.001
MH	0.148±0.010	0.158±0.009^a^	0.150±0.010^ab^	*(0.82)*	*(0.30)*	*(0.36)*
SH	0.152±0.010^1^,^2^	0.163±0.010^a^^1^,^2^	0.150±0.009^ab^			
**AERIAL TIME (S)**						
SL	0.095±0.010	0.096±0.011	0.094±0.010	0.026	0.583	0.886
MH	0.093±0.010	0.095±0.009	0.093±0.010	*(0.26)*	*(0.04)*	*(0.02)*
SH	0.094±0.010	0.096±0.011	0.093±0.010			
**STEP FREQUENCY (HZ)**						
SL	4.14±0.22	4.01±0.21^a^	4.11±0.24^b^	<0.001	0.039	0.002
MH	4.17±0.24	3.98±0.22^a^	4.14±0.25^b^	*(0.80)*	*(0.24)*	*(0.30)*
SH	4.07±0.22^1^,^2^	3.88±0.17^a^^1^	4.14±0.21^b^			
**STEP LENGTH (M)**						
SL	1.51±0.11	1.48±0.09	1.51±0.12	0.008	0.410	0.987
MH	1.49±0.11	1.47±0.10	1.50±0.11	*(0.33)*	*(0.07)*	*(0.01)*
SH	1.49±0.11	1.47±0.10	1.50±0.11			

*Mean ± SD (n = 13). The repeated-sprint exercise protocol included a first set of eight sprints performed at sea level (SL), moderate (MH), or severe hypoxia (SH), while the second set of four sprints was always performed at SL*.

a, b *Significant different from average of sprint 1–4 and 5–8, respectively (P < 0.05)*.

1, 2 *Significant different from SL and MH, respectively (P < 0.05)*.

### Surface EMG activity

Temporal profiles of the EMG amplitude (RMS) and frequency spectrum (MPF) for the six investigated muscles are shown in Tables 2, 3. With the exception of *Rectus femoris* RMS activity displaying lower values in SH compared to SL for sprint 8 (*P* < 0.05), all other investigated muscles RMS and MPF values fell significantly over time (*P* < 0.05), independently of the condition. The decrease in RMS activity from sprint 1 to 4, expressed as a percentage of sprint 1 value, was significant for *Vastus lateralis, Rectus femoris, Gastrocnemius lateralis*, and *Tibialis anterior* muscles (*P* < 0.05), while all muscles displayed lower values in all conditions during sprint 8 (Table 2). After 6 min of rest, a recovery in the RMS activity of all muscles (except for *Biceps femoris*) occurred during sprint 9, which was not statistically different than that in sprint 1. During sprint 12, RMS activities for all muscles were lower than those of sprints 4 (except for *Biceps femoris*) and 9 (*P* < 0.05). When compared to sprint 1 (100%), MPF values were reduced during sprint 8 for *Vastus lateralis* and *Rectus femoris*, during sprint 9 for *Gastrocnemius lateralis* and *Tibialis anterior* and during sprint 12 for *Biceps femoris* and *Gastrocnemius medialis* (Table 3; *P* < 0.05).

**Table 2:** **Surface EMG root mean square (RMS) activity**.

**Variables (% sprint 1)**	**Sprints**				**ANOVA *p*-value *(partial eta-squared)***		
	**4**	**8**	**9**	**12**	**Condition**	**Time**	**Interaction**
**RMS *vastus lateralis***							
SL	90.9±9.5^a^	85.0±17.0^a^	92.4±16.8^c^	86.1±17.2^a^^d^	0.987	<0.001	0.923
MH	89.2±11.7^a^	86.3±11.7^a^	94.3±9.4^c^	84.9±14.4^a^^d^	*(0.01)*	*(0.52)*	*(0.03)*
SH	88.8±11.8^a^	83.0±13.1^a^	96.1±5.8^c^	84.8±6.0^a^^d^			
**RMS *rectus femoris***							
SL	88.8±9.2^a^	84.8±9.1^a^^b^	98.1±11.7^c^	88.1±9.9^a^^d^	0.166	<0.001	0.036
MH	96.8±10.2^a^	85.5±13.3^a^^b^	94.6±14.2^c^	84.3±17.0^a^^d^	*(0.14)*	*(0.67)*	*(0.15)*
SH	84.1±10.6^a^	75.5±9.9^a^^b^^1^	91.1±13.7^c^	81.8±12.4^a^^d^			
**RMS *biceps femoris***							
SL	92.9±10.4	87.1±13.3^a^	94.7±9.0^a^	85.1±18.6^a^^b^^d^	0.906	<0.001	0.535
MH	98.9±9.2	91.6±9.2^a^	90.2±12.9^a^	85.4±15.3^a^^b^^d^	*(0.01)*	*(0.53)*	*(0.06)*
SH	93.3±9.4	89.2±9.5^a^	94.2±11.7^a^	85.6±15.5^a^^b^^d^			
**RMS *gastrocnemius medialis***							
SL	93.8±5.9^a^	86.2±10.2^a^^b^	99.6±9.9^c^	91.9±18.2^a^^c^^d^	0.336	<0.001	0.553
MH	93.2±11.2^a^	82.6±13.9^a^^b^	92.4±12.9^c^	84.5±18.9^a^^c^^d^	*(0.09)*	*(0.61)*	*(0.06)*
SH	90.3±9.7^a^	81.9±8.4^a^^b^	95.7±9.5^c^	91.5±11.6^a^^c^^d^			
**RMS *gastrocnemius lateralis***							
SL	94.5±12.0	86.8±10.4^a^^b^	98.0±8.8^c^	88.3±13.1^a^^d^	0.725	<0.001	0.657
MH	95.4±10.3	82.5±17.1^a^^b^	99.1±14.9^c^	87.4±13.6^a^^d^	*(0.02)*	*(0.63)*	*(0.05)*
SH	89.7±12.1	83.0±13.8^a^^b^	97.6±8.1^c^	88.2±13.4^a^^d^			
**RMS *tibialis anterior***							
SL	85.6±13.2^a^	72.5±13.9^a^^b^	98.4±10.8^c^	83.4±16.1^a^^d^	0.302	<0.001	0.039
MH	87.3±10.4^a^	84.4±15.5^a^^b^	96.6±10.8^c^	85.1±10.8^a^^d^	*(0.10)*	*(0.71)*	*(0.15)*
SH	83.9±13.3^a^	76.3±13.2^a^^b^	90.0±10.0^c^	80.5±13.7^a^^d^			

*Mean ± SD (n = 13). The repeated-sprint exercise protocol included a first set of 8 sprints performed at sea level (SL), moderate (MH), or severe hypoxia (SH), while the second set of 4 sprints was always performed at SL*.

a, b, c, and d *significant different from sprint 1, 4, 8, and 9, respectively (P < 0.05)*.

1 *significant different from SL (P < 0.05)*.

**Table 3:** **Surface EMG median power frequency (MPF)**.

**Variables (% sprint 1)**	**Sprints**				**ANOVA *p*-value *(partial eta-squared)***		
	**4**	**8**	**9**	**12**	**Condition**	**Time**	**Interaction**
**RMS *vastus lateralis***							
SL	96.1±10.0^a^	94.2±13.0	97.7±10.9	94.3±11.9	0.977	0.024	0.923
MH	95.4±13.0^a^	95.0±12.6	98.0±12.3	93.9±16.0	*(0.02)*	*(0.21)*	*(0.03)*
SH	99.2±8.4^a^	93.2±10.0	94.4±13.8	92.9±14.4			
**RMS *rectus femoris***							
SL	99.9±5.2^a^	96.4±7.6^a^^b^	98.8±8.6^c^	99.0±10.6	0.186	<0.001	0.167
MH	89.6±10.9^a^	88.8±8.8^a^^b^	97.5±11.6^c^	95.1±14.1	*(0.14)*	*(0.42)*	*(0.13)*
SH	94.1±10.3^a^	87.6±13.1^a^^b^	94.2±13.2^c^	89.3±16.4			
**RMS *biceps femoris***							
SL	96.2±7.2	95.9±10.9	90.0±16.4	90.1±18.7	0.208	0.046	0.509
MH	99.3±12.2	94.8±13.0	95.1±15.6	94.4±11.8	*(0.12)*	*(0.23)*	*(0.07)*
SH	105.1±6.1	101.7±10.8	94.2±11.2	97.1±14.9			
**RMS *gastrocnemius medialis***							
SL	98.7±9.3	99.4±9.2	92.9±12.3	93.3±8.8^a^	0.886	0.027	0.341
MH	94.8±7.4	92.7±10.6	96.0±11.1	95.2±11.5^a^	*(0.01)*	*(0.20)*	*(0.09)*
SH	97.8±10.0	97.4±10.9	95.0±9.8	94.3±10.0^a^			
**RMS *gastrocnemius lateralis***							
SL	96.8±5.1	99.9±9.3	95.1±7.7^a^	98.9±12.2	0.264	0.011	0.607
MH	97.4±6.3	94.4±11.5	94.9±12.6^a^	94.1±8.3	*(0.11)*	*(0.23)*	*(0.06)*
SH	95.8±9.4	94.6±10.7	91.7±12.3^a^	91.2±11.2			
**RMS *tibialis anterior***							
SL	97.2±9.3	99.7±10.8	96.3±6.9	98.2±11.5	0.663	0.011	0.605
MH	97.5±5.9	94.5±10.1	96.3±15.8	93.3±13.9	*(0.03)*	*(0.23)*	*(0.06)*
SH	97.9±5.6	95.7±11.0	91.8±8.4	96.7±6.5			

*Mean ± SD (n = 13). The repeated-sprint exercise protocol included a first set of 8 sprints performed at sea level (SL), moderate (MH) or severe hypoxia (SH), while the second set of 4 sprints was always performed at SL*.

a, b, and c *significant different from sprint 1, 4, and 8, respectively (P < 0.05)*.

## Discussion

### Different levels of acute hypoxia alter RSA and neuro-mechanical adjustments

SpO_2_ values were increasingly lower as O_2_ availability decreased, yet cardio-vascular (HR) and perceptual (RPE) loads associated with performing repeated treadmill sprints were not incrementally higher, which may be due in part to the lower work performed at SH and the “all out” nature of the present exercise (Balsom et al., 1994). Hence, fatigue-induced decrement in sprint distance was significantly exacerbated in SH relative to SL, while sprint performance was relatively resilient to MH exposure. Single (i.e., sprint 1 in the present study) sprint performance is known to be unaffected by differing hypoxia levels (Billaut et al., 2013). For instance, treadmill sprint performance for efforts lasting 60 s or less is not adversely affected at altitude (FiO_2_ = 13%) (Weyand et al., 1999). This may relate to an enhanced anaerobic energy release to compensate for the reduced aerobic ATP production (Calbet et al., 2003; Ogawa et al., 2007). However, earlier and larger performance decrements usually occur when consecutive sprints are performed in O_2_-deprived environments with hypoxia-related effects becoming more evident above 3000 m (Bowtell et al., 2014; Goods et al., 2014).

During set 1 of all trials, the temporal aspects of the stride cycles shifted toward an increase in contact and aerial times, along with reductions in step frequency. Collectively, it demonstrates a deteriorated ability to tolerate ground impact/stretch loads as fatigue develops with sprint repetitions. In line with these findings, similar impairments in sprint kinematics have been connected with progressively slower sprint performance during over-ground [i.e., 6 × 20 m – 20 s of passive recovery in U19 footballers (Girard et al., 2011a); 6 × 35 m –10 s of passive recovery in elite footballers (Brocherie et al., 2015); 12 × 40 m –30 s of passive recovery in team- and racquet-sports athletes (Girard et al., 2011b)] or treadmill [i.e., 5 × 5-s sprints–25 s of passive recovery (Girard et al., 2015a); 3 sets of 5 × 6-s sprints–24 s of passive recovery between sprints and 3 min between sets (Morin et al., 2011) in athletes with a team-sport background] repeated sprints. Furthermore, the larger magnitude of repeated-sprint performance alterations seen at SH compared to SL and MH was due to exacerbated increases in contact time and decreases in step frequency in the severer hypoxic condition. Slower sprints and less efficient stride characteristics in SH compared to SL or MH appear to be the result of individuals applying less forward-oriented forces. In line with previous literature (Morin et al., 2011), our primary biomechanical rationale for this conclusion is based on the fact that the magnitude of reductions for horizontal forces was three times larger than for resultant (total) forces.

Remarkably, most of the alteration in performance and accompanying running mechanics was observed within the first half of the first set (sprints 1–4) with smaller changes during the second part (sprints 5–8). During the completion of ten, 10-s sprints with 180 s of recovery the rate of decline in total work was also greater during the first 5 sprints compared to the last 5 sprints (−5.2% vs. −3.3%) (Pearcey et al., 2015). In this later study, neuromuscular fatigue in the first 5 sprints was mainly peripheral, whereas in the last 5 sprints it was both peripheral and central. By assessing the development of fatigability during repeated-sprint running exercise (12 × 30 m–30 s rest), it has also been reported that significant peripheral and central knee extensor fatigue becomes evident after just two maximal sprints (Goodall et al., 2015). In our study, the etiology of neuromuscular fatigability (i.e., using peripheral and/or magnetic stimulations) during or after repeated sprinting has not been specifically investigated. Using such stimulation procedure and exposure to acute moderate hypoxia (i.e., FiO_2_ = 13.8%; Billaut et al., 2013) or the induction of pre-existing locomotor muscle fatigue (i.e., following a 10-min neuromuscular electrical stimulation protocol of the quadriceps; Hureau et al., 2014) it was, however, evidenced that feedbacks from fatiguing muscles play an important role in the determination of central motor drive and force output during RSA protocols; i.e., the development of peripheral muscle fatigue would be confined to a certain level so as not to surpass a sensory tolerance limit.

During the first repeated-sprint set, RMS activity values of all investigated muscles decreased significantly over time, confirming that neural factors may have played a role in fatigue-related decrement in sprint performance (Bowtell et al., 2014; Brocherie et al., 2015). This emphasis a decreased number of motor units activated and/or firing rates of the recruited motor units in exercising quadriceps and plantar flexor muscles, yet with no possible distinction between these two phenomena. Our results also feature an earlier and larger central down-regulation of skeletal muscle recruitment in SH compared to SL or MH, even though this observation is restricted to the *Rectus femoris* muscle only. Exacerbated performance decrements under severe hypoxia are likely to be explained by a reduced neural drive to the active musculature, arising secondary to a stronger reflex inhibition due to brain hypoxia (i.e., decreased brain oxygenation independently of afferent feedback and peripheral fatigue; Millet et al., 2012a) or a hypoxia-induced increased level of intramuscular metabolites known to stimulate group III-IV muscle afferents (Hogan et al., 1999). Although, hypoxia exposure would exacerbate exercise-induced demand placed upon the central nervous system to explain premature fatigue, it is important to emphasize that local metabolic factors (not measured here) may also be responsible for the greater fatigue incurred in SH vs. other conditions.

Reductions in MPF during exercise are indicative of a slowdown of muscle fiber action potential conduction velocity (Lindstrom et al., 1970). In the present study, the values of MPF from the *Vastus lateralis* and *Rectus femoris* muscles during sprint 8 were significantly lower than in sprint 1, while there was no condition effect. This result differs from that of Matsuura et al. (2006) who suggested, based on lower MPF values during repeated cycling sprints with 35-s vs. 350-s recovery periods, that a severer metabolic state (i.e., increased hypoxia severity) induces preferred recruitment of slow twitch motor units.

### Restoration of sprint mechanical performance between repeated-sprint sets

Conceivably, perceptual recovery, which is known to interact with both feed-forward/feed-back mechanisms, may well affect athlete's willingness to maintain maximal efforts during successive sprint actions. Despite distance covered and resulting HR not being different, RPE values were elevated during the second compared to the first repeated-sprint set at similar time points (sprint 9 vs. 1 and 12 vs. 4). This indicates that perception of peripheral discomfort may not be the major performance regulator during RSA running protocols. Also in line with this assumption are the well-preserved quadriceps muscle activation and associated power output that occurred during two 4-s maximal cycling bouts under hypoxic (FiO_2_ = 13%) and normoxic conditions, despite higher overall perceived peripheral discomfort and perceived difficulty breathing (Christian et al., 2014). Nevertheless, the role of effort perception during recovery should not be disregarded as, for instance, the magnitude of the core temperature decrease and the subjective perception of recovery following cold water immersion after an intense conditioning session have been related to performance enhancement in a repeated 40 m sprint protocol undertaken 24 h later (Cook and Beaven, 2013). Nonetheless, future studies should isolate perceptual responses to recovery as mitigating of improved performance.

After the 6 min of passive rest between sprint 8 and sprint 9, the temporal aspects of the stride cycle (contact and aerial times, step frequency and step length values) and force production characteristics (mean horizontal and resultant forces) during sprint 9 recovered from those recorded during sprint 8 and were not different from sprint 1. When physical education students performed four sets of five, 6-s sprints (24 s of passive recovery between sprints, 3 min of rest between sets), Morin et al. (2011) observed that the level of performance was almost systematically higher at the beginning of sets 2, 3, and 4 than at the end of sets 1, 2, and 3. Thus, to preserve RSA performance it is practically important to apply large forward-oriented total force against the ground and minimize the decrease in step frequency (i.e., increase in contact time). Furthermore, despite differing hypoxic severity levels during sprints 1–8, distance covered as well as the main kinetic and kinematic variables measured at sprint 9 were restored near sprint 1 in all conditions. Interestingly, SpO_2_ values recorded for sprint 9 were similar to those of sprint 1 in the SL condition, with also no difference between conditions for the average of the four sprint repetitions of the second normoxic set. In fact, restoration of SpO_2_ levels near baseline SL values is virtually complete after 6 min of normoxic exposure. Collectively, it shows that hypoxia level of an initial sprint bout may not blunt the post-exercise recovery of single and repeated-sprint performance and its mechanical basis.

Restoration of EMG indices (RMS and MPF values) appear to align with sprint mechanical performance recovery between sprints 8 and 9, with no difference when comparing initial efforts of the two repeated-sprint sets (sprints 1 vs. 9). This reinforces that the ability to fully activate the contracting musculature and/or optimal inter-muscle recruitment strategies are important regulators of RSA. These results, however, are in disagreement with those reporting that EMG amplitude remained depressed (~12%), after 6 min of rest, during the initial repetition of the second exercise set, despite mechanical performance being matched for first sprint of the two repeated-sprint series (Mendez-Villanueva et al., 2007). Compared to normoxia, cycling performance and quadriceps muscle activation during a multiple sets RSA protocol (three sets of five 5-s cycling sprints with 25 s of passive recovery between sprints and 120 s of rest between sets) was lower in moderate hypoxia (FiO_2_ ~0.14), with also incomplete apparent recoveries of performance between the last repetition of sets 1 and 2 (i.e., sprints 5 and 10) and the initial repetition of sets 2 and 3 (i.e., sprints 6 and 11) (Billaut et al., 2013). Accordingly, it is difficult to directly compare our results with other experimental/environmental conditions. In the present study, restoration of sprint mechanical performance following prior repeated sprints at differing hypoxia severity resulted from the recovery of muscle recruitment patterns, which implicates a role for central mechanisms in the regulation of post-exercise recovery. However, the role of peripheral recovery should not be overlooked since it was demonstrated, using a similar exercise protocol, that phosphocreatine re-synthesis was associated with total work done during the first sprint of the second set (*r* = 0.79, *P* < 0.05) and total work done during the five sprints of the second set (*r* = 0.67, *P* < 0.05) (Mendez-Villanueva et al., 2012).

### Hypoxia has no residual effect during a subsequent normoxic repeated-sprint performance

An important determinant of fatigue during repeated sprinting is the initial (i.e., first sprint) mechanical output, which has consistently been positively correlated with performance decrement over subsequent sprints (Girard et al., 2011c). In this study, similar performance was observed during initial sprints of both sets with no difference between conditions. Furthermore, the averaged values of distance covered, kinetics and kinematics for the four sprints of the second set (i.e., sprints 9–12; fatigued muscles) were similar across conditions and were not statistically different in reference to the average of the first four sprints of the initial set (i.e., sprints 1–4; non-fatigued muscles). With this in mind, our results indicate that recent muscle activation (completion of the first set) does not alter the muscle recruitment pattern and fatigability during a second set of repeated sprints completed near sea level after a 6 min (normoxic) resting period. These results contrast with those of Mendez-Villanueva et al. (2007) who indicated that after 6 min of rest following 10, 6-s cycling sprints, participants were able to reproduce during sprint 11 the mechanical performance achieved during sprint 4, but not RSA. In the above study, greater fatigability was evident in the five repetitions of the second (i.e., sprints 11–15) vs. the first set (i.e., sprints 4–8), suggesting different recovery time courses after single sprint and RSA performances. Despite severer hypoxia levels during a first repeated-sprint exercise bout, majoring exercise-induced demands placed on the neuromuscular system (i.e., contact times and step frequencies for sprints 5–8 differed from sprints 1 to 4 and 9 to 12), there was no apparent fatigue-related residual or “carry-over” effects from this previous set. Hence, RSA was similar across conditions during the completion of a second set of normoxic repeated sprints.

With different exercise-to-rest ratios influencing, to a large extent, the oxidative vs. glycolytic component (Tabata et al., 1997), RSA may not be similarly affected by hypoxia exposure, which complicates comparison of our results with those of previous studies. Although, evidence is currently lacking, it is anticipated that narrower exercise-to-rest ratios (1:2–1:4 vs. 1:5 as used here) and severer hypoxic conditions, inducing a decreased O_2_ availability and an increased reliance on O_2_-independent glycolysis for ATP resynthesis together with a larger recruitment of fast-twitch fibers, may exacerbate sprint performance decrements. While similar blood lactate concentration levels observed here may suggest otherwise, whether glycolytic vs. aerobic contributions actually differed between our three conditions would need to be confirmed from muscle oxygenation, phosphocreatine metabolism and/or pH recordings. RSA protocols using different exercise-to-rest ratios and hypoxia levels in the same group of participants would also be helpful in this instance. The resting period duration between the two sets is obviously a key parameter for any type of multiple repeated-sprint sets. In the “hypoxia-to-normoxia recovery” protocols, this duration is paramount as it determines SpO_2_ levels at the start of the second set. In this study, SpO_2_ values measured 4 min into recovery between the two repeated-sprint sets returned near baseline and did not differ between conditions. It is, however, likely that SpO_2_recovery to initial values was even shorter. Hence, Krivoshchekov et al. (2014) reported that the SpO_2_ recovery response after an acute exposure to normobaric hypoxia (FiO_2_ = 0.10) decreasing SpO_2_ to 85% was ~120 s.

### Limitations

Before concluding, we must acknowledge several limitations that may affect generalization of our findings. Firstly, although the data were collected continuously (step-by-step), our analysis was concentrated on 3 steps at top speed (i.e., usually corresponding to the 20 m mark of the sprints). It is noteworthy, however, that considering all steps or only a few steps during early, middle or late phases of 5-s sprints provides similar mechanical outcomes during repeated treadmill sprinting, although acceleration induces noticeable differences between the sections studied (Girard et al., 2015a). It must also be appreciated that running speed reached on our treadmill is 15–20% slower than over-ground, even though changes in running mechanics are relatively similar (Rabita et al., 2015) and overall sprint performance is highly correlated between these two sprinting modes (Morin and Sève, 2011). Although, an effect on performance induced by EMG electrodes or mask breathing cannot be completely ruled out, the use of wireless technology and the fact that resistance and increase in dead space are negligible (Sheel, 2002) suggests that their influence did not modify the main findings of the present study.

Secondly, several concerns may affect EMG analysis and include: (1) surface EMG amplitude cancellation; (2) the stability of neuromuscular propagation and sarcolemmal excitability (i.e., absence of supra-maximal stimulation to evoke a M-wave for normalization of the EMG signal); (3) fatigue-related reflex inhibition (i.e., reflex effects in the spinal cord). With differences < 5%, the surface EMG signal may not be sufficiently sensitive to measure meaningful (i.e., clinically relevant) difference in muscle activation between conditions. Therefore, declines in the magnitude of efferent descending motor outflow, as a key factor in neuromuscular recovery following repeated sprints, would need to be confirmed through the use of multiple neurophysiological measures (TMS, EEG) during resting intervals. The kinetics of muscle oxygenation (NIRS) would also be valuable for better comparing the metabolic differences between hypoxic conditions.

Thirdly, we have used three known values of FiO_2_ as hypoxic stimulus. For exposure to the same simulated altitude (FiO_2_ = 10%), however, it is conceded that there is a larger inter-individual variability in the degree of arterial hypoxemia compared to clamped values of SpO_2_ (at 75%) (Hamlin et al., 2010). While clamping of SpO_2_ would likely cause a more consistent hypoxic stimulus across individuals, it remains to be demonstrated that it will also induce a better heterogeneity in neuro-mechanical responses to repeated sprinting. Furthermore, hypobaric hypoxia has been shown to induce severer physiological responses (SpO_2_ and HR) than normobaric hypoxia (Millet et al., 2012b). One may therefore speculate that the performance and mechanical alterations would be larger at natural altitude than in the present laboratory study. Direct comparisons of repeated sprint exercises between normobaric and hypobaric hypoxia are required.

Finally, in our study, we implemented 6 min of rest between the two sets of repeated treadmill sprints, so as to compare our results with previous findings (Mendez-Villanueva et al., 2007, 2012). However, not only are the acute neuro-mechanical adjustments and the ensuing recovery of SpO_2_ and performance influenced by the duration/nature of the between-sets normoxic rest period but also the details of the RSA protocols (e.g., exercise-to-rest ratio, exercise mode, environment encountered; Girard et al., 2011c) and participants' background (e.g., training status, “aerobic” vs. “anaerobic” profile, gender; Calbet et al., 2003). Given the task-dependency of the effects of fatigue, our conclusions must remain specific to the circumstances of this study and would need to be confirmed using other RSA protocols and participants.

## Conclusion

To improve our understanding of neuro-mechanical determinants of RSA, we manipulated the hypoxia severity during an initial set of repeated sprints and examined the effect on alterations in performance, running mechanics and lower-limbs neuromuscular activity during a subsequent set completed in normoxia. Under the circumstances of this study (participants' background, exercise-to-rest ratio, hypoxia exposure), the magnitude of performance and neuro-mechanical alterations (kinetics, kinematics, EMG indices) and the severity of physiological and perceptual responses were larger in SH compared to SL and MH. The novel findings from our “recovery of performance” approach are that recoveries of performance and neuro-mechanical alterations are almost complete after resting for 6 min near sea level, with also a similar fatigue pattern across conditions during subsequent repeated sprints in normoxia. To preserve RSA performance, it is therefore important to apply large forward-oriented ground reaction force and minimize the decrease in step frequency (i.e., increase in contact time), which at least in part result from more optimal neural drive strategies. However, no singular factor may represent a direct causative mechanism determining RSA so that studying the potential for other drivers of recovery (e.g., muscle damage or metabolic factors) may also be relevant.

## Author contributions

Conceived and designed the experiments: OG and FB. Performed experiments: OG and FB. Analyzed data: OG, FB, and JM. Interpreted results of research: OG, FB, JM, and GM. Drafted manuscript and prepared tables/figures: OG. Edited critically revised paper and approved final version of manuscript: OG, FB, JM, and GM.

## Funding

This work is based on research funded by QNRF (NPRP 4 – 760 – 3 – 217).

### Conflict of interest statement

The authors declare that the research was conducted in the absence of any commercial or financial relationships that could be construed as a potential conflict of interest.
